# KLK7 Involvement in Thyroid Papillary Carcinoma Cell Migration and Invasion by EMT via MAPK/ERK Pathways

**DOI:** 10.7150/jca.101555

**Published:** 2025-02-11

**Authors:** Min Li, Zi-Wen Li, Jia-Yin Song, Yu Bin, Tao Ni, Gang Xue, Xu Lin, Jing-Fang Wu

**Affiliations:** 1 Department of Morphology Laboratory, Hebei North University, Zhangjiakou, 075000, China.; 2 Department of Otorhinolaryngology-Head and Neck Surgery, The First Affiliated Hospital of Hebei North University, Zhangjiakou, 075000, China.

**Keywords:** KLK7, PTC, TCGA, MAPK/ERK signaling, EMT

## Abstract

**Purpose:** KLK7, also known as Kallikrein 7, is a secreted enzyme classified as a serine protease. Earlier studies have indicated that KLK7, KLK10, and KLK11 are linked to the survival rates and immune reactions of individuals with papillary thyroid cancer (PTC). This research examines KLK7, investigating its role and expression, and evaluates its viability as a treatment target for PTC.

**Methods:** Initially, we examined the expression and possible functions of KLK7 in PTC using bioinformatics techniques. Researchers examined the impact of KLK7 on the cancer characteristics of PTC and explored if KLK7 influences the Epithelial-mesenchymal transition (EMT) process via the MAPK/ERK pathway in PTC using methods like immunohistochemistry and growth curve analysis. Ultimately, a model using a nude mouse was conducted to confirm the impact of KLK7 on PTC.

**Results:** Our research demonstrated that KLK7 exhibited variations in THCA tissues, and KLK7-related genes had the role of participating in protein synthesis, genetic variation, mRNA degradation and immune microenvironment of PTC. KLK7 was upregulated in PTC tissues and positively associated with clinical stage and lymph node metastasis. Furthermore, the inhibition of KLK7 significantly diminished the proliferation, migration, and invasiveness of PTC cells. Notably, silencing KLK7 reduced phosphorylation of ERK1/2 and suppression of EMT. *In vivo* experiments further supported these findings. KLK7 might serve as an efficacious therapeutic target and predictive biomarker for PTC patients.

**Conclusion:** KLK7 could be essential in the cancerous advancement of PTC by influencing the EMT via the MAPK/ERK signaling pathway, thereby impacting the growth, migration, and invasiveness of PTC cells. KLK7 appears to be a promising candidate for targeting in PTC therapy.

## 1. Introduction

Papillary thyroid cancer (PTC) is the most prevalent type of thyroid cancer, accounting for approximately 80% of all cases 1. Recent advancements in diagnostic techniques have significantly improved the detection and characterization of PTC, leading to more precise prognostic assessments and more effective treatment strategies 2, 3. Despite these advancements, the recurrence and progression of PTC remain clinical challenges, highlighting the need for improved understanding of its molecular mechanisms 4, 5. A critical area of ongoing research involves the investigation of molecular markers that can predict disease behavior and therapeutic response. In this context, the Kallikrein-related peptidase (KLK) family has emerged as a potential key player. Although multiple studies have pointed to the abnormal expression of various KLKs in different malignancies, their specific roles in PTC progression and immune response modulation are still insufficiently characterized 6.

The KLK family, consisting of 15 serine proteases, plays a role in several regulated functions, such as skin shedding, semen liquefaction, and the regulation of blood pressure 7-9. Beyond these roles, KLKs have been implicated in cancer pathology, primarily through mechanisms related to tumor microenvironment modulation, cell migration, and invasion 6, 10. In the context of thyroid cancer, research has demonstrated that KLK7, KLK10, and KLK11 are predictors of poor outcomes and are often associated with more severe tumor grades and later stages of the disease 11, 12. These enzymes are believed to influence cancer progression through the degradation of extracellular matrix components, thereby facilitating tumor cell dissemination and metastasis 13. However, the literature reveals a gap in the targeted exploration of KLK7 within PTC, particularly in relation to its regulatory mechanisms, and direct impact on cancer cell behavior.

Based on preliminary bioinformatics analyses indicating an association between high KLK7 expression and reduced survival and increased immune infiltration in PTC, this study seeks to deepen the understanding of KLK7's functional roles in PTC pathology 11. While earlier studies have established a broad link between KLKs and cancer progression 12-14, few have specifically identified the mechanistic pathways through which KLK7 influences PTC progression. This research employed public databases and immunohistochemical tissue microarrays from cancer specimens to examine the differential expression levels of KLK7 in malignant versus healthy tissues, revealing its significance for clinical staging and lymph node metastasis. Additionally, we confirmed KLK7's role in regulating cell growth, migration, and invasion by conducting *in vitro* experiments with PTC cell lines. Furthermore, we conducted subcutaneous tumorigenesis experiments in nude mice to verify the *in vivo* expression of KLK7. Consequently, our evaluation indicates that KLK7 could be a dependable indicator for forecasting unfavorable tumor outcomes and reactions to immunotherapy. Nonetheless, the possible molecular pathways leading to increased KLK7 levels during cancer development need more fundamental and clinical studies for investigation.

## 2. Materials and Methods

### 2.1. Differential expression and mutation analysis of KLK7

The expression levels of KLK7 in both matched and unmatched samples were obtained from RNA-seq data provided by the TCGA thyroid cancer (THCA) project and the GTEx repository. Information on KLK7 mutations in THCA was sourced from the COSMIC database (https://cancer.sanger.ac.uk). "Thyroid" was selected under "Tissue Distribution" and "Mutation Distribution". Clinical features of THCA patients were obtained from the TCGA THCA project to analyze their correlation with KLK7 expression in THCA samples. Furthermore, a logistic regression analysis was conducted to assess the relationship among THCA's clinical characteristics and KLK7, accompanied by a forest plot visualization.

### 2.2. Analysis of KLK7 gene expression differences

Using the DESeq2, a differential analysis focusing on the KLK7 gene within the THCA project was conducted. THCA samples were divided into high and low KLK7 expression groups, with the median expression level serving as the threshold. A volcano plot was generated using the ggplot2 package to visualize the results of the differential gene analysis. ∣LogFC∣ > 1 and adjusted P-value < 0.05 were considered as the screening criteria for differentially expressed genes (DEGs). Cytoscape software was employed to examine the protein-protein interactions (PPI) of DEGs, and genetic status were subsequently recognized using the MCODE extension. Ultimately, genes were ranked from highest to lowest according to their 'Pearson value' from the single-gene correlation study, and the top 29 most correlated genes were selected. Utilizing the ggplot2 package, a heatmap showcasing the co-expression of the KLK7 gene with the top 50 genes, including members of the KLK gene family (KLK1-15), was generated.

### 2.3. Functional enrichment analysis

To investigate possible signaling pathways, gene set enrichment analysis (GSEA) was employed, focusing on the differential expression analysis, comparing samples exhibiting high KLK7 expression against those manifesting low KLK7 expression. The reference gene set utilized was c2.cp.all.v2022.1.Hs.symbols.gmt. Based on the criteria for differentially expressed genes (|LogFC| > 1 and modified P-value < 0.05), Gene Ontology (GO) and Kyoto Encyclopedia of Genes and Genomes (KEGG) pathway analyses were performed to identify pathways associated with KLK7 in thyroid cancer, using 'clusterProfiler,' 'msigdbr,' and 'org.Hs.eg.db' packages.

### 2.4. Identification of immune infiltration in THCA samples with different KLK7 expression levels

To assess immune infiltration for the TCGA THCA project, the ssGSEA method from was employed 15. And the association between KLK7 expression and major immune cells and immune molecules was visualized using the ggplot2 package 16.

### 2.5. Patient specimens

The PTC tissue microarray, identified by batch number HThy-Can060PT-01, was acquired from Shanghai Superchip Biotechnology Co., Ltd. It includes 28 cases of thyroid cancer and paraneoplastic tissue, and 4 cases of normal thyroid tissue. And samples preserved in paraffin were acquired from the First Affiliated Hospital of Hebei North University, encompassing 10 instances of PTC identified from January 2020 to December 2023. The Ethics Committee at the First Affiliated Hospital of Hebei North University granted approval for this research. The participants were notified and consented to join the research. The eligibility criteria are as follows: (1) Initial consultation at the Department of Otorhinolaryngology-Head and Neck Surgery, First Affiliated Hospital of Hebei North University; (2) Histopathological diagnosis of papillary thyroid carcinoma (PTC); (3) Consent to undergo thyroidectomy (either total or partial) and lymph node dissection; (4) Availability of complete preoperative laboratory results, electronic medical records, and postoperative pathology reports. Among the 38 cases of thyroid cancer, 11 were male and 27 were female, ranging in age from 29 to 79, with an average age of 56.2 ± 11.5 years.

### 2.6. Immunohistochemistry experiment

Tissue microarrays were routinely deparaffinized to water and then washed with 0.01 M PBS. Antigen retrieval was conducted using a 0.01 M citrate buffer solution. Endogenous peroxidase activity was blocked by incubating the samples with a natural peroxidase inhibitor at room temperature for 10 minutes. The primary antibody, KLK7, was diluted at a ratio of 1:80 and incubated overnight at 4°C. The enzyme-tagged goat anti-mouse/rabbit IgG polymer, functioning as the secondary antibody, was left to incubate at 37°C for half an hour. DAB staining was used for visualization, hematoxylin for counterstaining the nuclei, and the slides were dehydrated, clarified, and mounted with neutral balsam. Observations were made using an Olympus BX-51 optical microscope.

### 2.7. Cell culture and transfection

Cells including TPC-1, BCPAP, K1, and Nthy-ori 3-1 were sourced from the Shanghai Institute for Biological Sciences, Chinese Academy of Sciences (ATCC), and cultured in RPMI 1640 complete medium, sustained at 37°C and 5% CO_2_. The culture medium was replenished every 48 hours. Cells in the exponential growth phase were then used for subsequent experiments. Cells were seeded in six-well plates and grown until they reached 70%-80% confluence. Transfection was carried out following the manufacturer's instructions for Lipofectamine 2000. The study was segmented into three groups: (1) Blank control group (K1/BCPAP group) with no treatment; (2) Negative control group (KLK7-shRNAC group) transfected with KLK7-NC; (3) Experimental group (KLK7-shRNA1 / shRNA2 / shRNA3 group) transfected with KLK7-shRNA1 / shRNA2 / shRNA3. Cells from each group were harvested 48 hours post-transfection to evaluate the efficiency using real-time quantitative PCR (RT-qPCR) and western blot (WB). The target sequences for KLK7-shRNA1 are 5'-CAAGTGGATAAATGACACCAT-3', for KLK7-shRNA2 are 5'-GCTGTCATCCATGGTGAAGAA-3', and for KLK7-shRNA3 are 5'-GACCCATGTTAATGACCTCAT-3'. The control KLK7-shRNAC sequence is 5'-CCTAAGGTTAAGTCGCCCTCG-3'.

### 2.8. RT-qPCR

RNA was extracted from cells and tissues using TRIzol, quantified, and reverse-transcribed into cDNA for RT-qPCR with fluorescence detection. β-actin served as the internal reference. The RT-qPCR protocol included an initial denaturation at 95°C for 30 seconds, followed by 40 amplification cycles of 95°C for 10 seconds and 60°C for 30 seconds. The comparative expression levels were determined using the 2(-ΔΔCT) technique. Supplementary Table 1 presents the primer sequences.

### 2.9. Western blot

Proteins were isolated from tissues or cells utilizing the Total Protein Extraction Kit. Protein concentrations were measured using the BCA assay, with 30 μg of total protein from each sample loaded onto a 12% SDS-polyacrylamide gel for analysis. Subsequently, the proteins were transferred to PVDF membrane. The membrane was blocked at room temperature for one hour, rinsed with PBST, and then incubated with primary antibodies overnight at 4°C: KLK7 (1:1000), ERK1/2 (1:1000), phosphorylated ERK (1:900), MMP2 (1:900), MMP9 (1:900), E-cadherin (1:900), N-cadherin (1:900), β-catenin (1:800), Vimentin (1:900), Snail (1:900), Slug (1:900), Twist (1:900), and GAPDH (1:10000). And HRP-conjugated goat anti-mouse/rabbit IgG (1:10000) was incubated at room temperature for 1.5 h. Protein bands were visualized using an ECL chemiluminescence reagent and captured with an OmegaLumW chemiluminescence imaging system (Aplegen, USA).

### 2.10. Immunocytochemistry experiment

The cell concentration in each group was adjusted to 1 × 10⁷ cells/mL and then added onto coverslips. When the cells achieved 80% confluence, they were washed with PBS and then fixed in 4% paraformaldehyde for a quarter of an hour. The cells underwent three washes with PBS, each lasting 3 minutes, followed by a 20-minute permeabilization using Triton X-100, and were then washed 3 more times with PBS. Cells were treated with 3% H_2_O_2_ for 20 minutes at room temperature. A suitable dilution of primary antibody KLK7 (1:1000) was incorporated. Following 3 PBS washes, a biotin-labeled secondary antibody solution (HRP-linked streptavidin, SP goat anti-mouse/rabbit streptavidin universal type) was introduced and left to incubate at 37°C for 60 minutes; subsequently, the cells underwent another three PBS washes, each lasting 3 minutes. DAB was used for staining development, followed by rinsing with running water. The cells were then counterstained with hematoxylin for 2 minutes, differentiated using hydrochloric acid ethanol, and blued with ammonia water. Subsequently, the cells were dehydrated through a graded series of ethanol solutions, cleared in xylene, and mounted with neutral resin for microscopic examination. The negative control used PBS instead of the primary antibody.

### 2.11. Growth curve to assess cell proliferation vitality

Cells were treated with 0.25% trypsin, resuspended, and then cells were resuspended at a concentration of 1 × 10⁷ cells/mL for each group. Then introduced 150μL RPMI 1640 Medium into the 96-well plate, with three duplicate wells for each group. The cells were cultured and imaged using the IncuCyte ZOOM long-term dynamic cell imaging system.

### 2.12. Colony formation

Cells from the four groups (KLK7-shRNA1, KLK7-shRNA2, and KLK7-shRNAC) were treated with 0.25% trypsin, then resuspended in RPMI 1640 complete medium to generate a single-cell suspension. Six-well plates were used to seed 400 cells from each group, and they were cultured until colonies could be seen without a microscope. Subsequently, the plates were taken out, rinsed thrice with PBS, fixed in 4% paraformaldehyde for a quarter-hour, stained with 1 mL of Giemsa for 20 minutes, washed under flowing water, and allowed to air dry. Colonies containing over 50 cells were counted.

### 2.13. Wound healing assay

Cells from the KLK7-shRNA1, KLK7-shRNA2, and KLK7-shRNAC groups were seeded in a 96-well plate with three replicate wells. When cells reached 90% confluence, wounds were created using a 96-well Wound Maker Tool. Detached cells were removed by washing, and the plates were returned to the incubator for continued culture. Images were captured at 0, 24, and 48 hours using the IncuCyte ZOOM. The migration rate was assessed and analyzed utilizing ImageJ software.

### 2.14. Transwell invasion assay

Cells from three different groups underwent a 24-hour serum starvation, were treated with trypsin for digestion, and then resuspended in a medium devoid of serum to achieve a cell focus of 1 × 10^7^ cells/mL. Matrigel matrix gel was added to serum-free RPMI 1640 medium, and 100 μL of the diluted mixture was positioned within the superior chamber of a transwell insert. Subsequently, 200 μL of the cell mixture was placed in the superior compartment, while 1 mL of RPMI 1640 with 10% FBS was introduced. The transwell insert was kept at 37°C for 48 hours. Following this, the liquid in the upper chamber was extracted, and a cotton swab was carefully used to remove the cells that hadn't traversed the membrane. The cells were fixed with 4% paraformaldehyde for 20 minutes, rinsed twice, stained with 0.1% crystal violet for 5 minutes, washed three times, and five randomly selected high-magnification fields were captured under a microscope to examine and count the cells.

### 2.15. Subcutaneous xenograft model

Mouse experiment was performed in compliance with the guidelines sanctioned by the Institutional Animal Care and Use Committee (IACUC) of SPF (Beijing) Biotechnology Co. Ltd. Female nude mice with an average weight of 20 grams and aged 5 weeks were obtained from SPF (Beijing) Biotechnology Co. Ltd. To assess the effect of KLK7 on cellular proliferation *in vivo*, 1 × 10^6^ control or KLK7-silenced K1 and BCPAP cells were subcutaneously injected into the axillary region of the right forelimb of each mouse. Tumor diameter and weight were measured on days 3, 6, 9, 12, 16, 20, and 22. The size of the tumor was determined by the equation: the volume was calculated as (length) × (width)^2 / 2. After 28 days, *in vivo* imaging of the mice was performed. The mice were then sacrificed, and the tumors were excised, weighed, and photographed for further analysis. The tumors were lysed, and total RNA was isolated using the TRIzol technique for RT-qPCR to assess KLK7 and EMT-related mRNA levels. Additionally, total proteins were extracted for WB analysis to ascertain the levels of KLK7, ERK pathway proteins, and EMT-related proteins.

### 2.16. Statistical analysis

The presented data are expressed as the mean ± standard deviation. All statistical analyses were performed using SPSS. The differences among the groups were assessed using ANOVA followed by Dunnett's post hoc test. Correlations between the immunohistochemical and clinical features of the tissue microarray were analyzed using Fisher's exact test. P < 0.05 was considered significant. All experiments were independently replicated three times.

## 3. Results

### 3.1. The potential association of KLK7 with the incidence of THCA and variations in THCA

**Figure 1:**
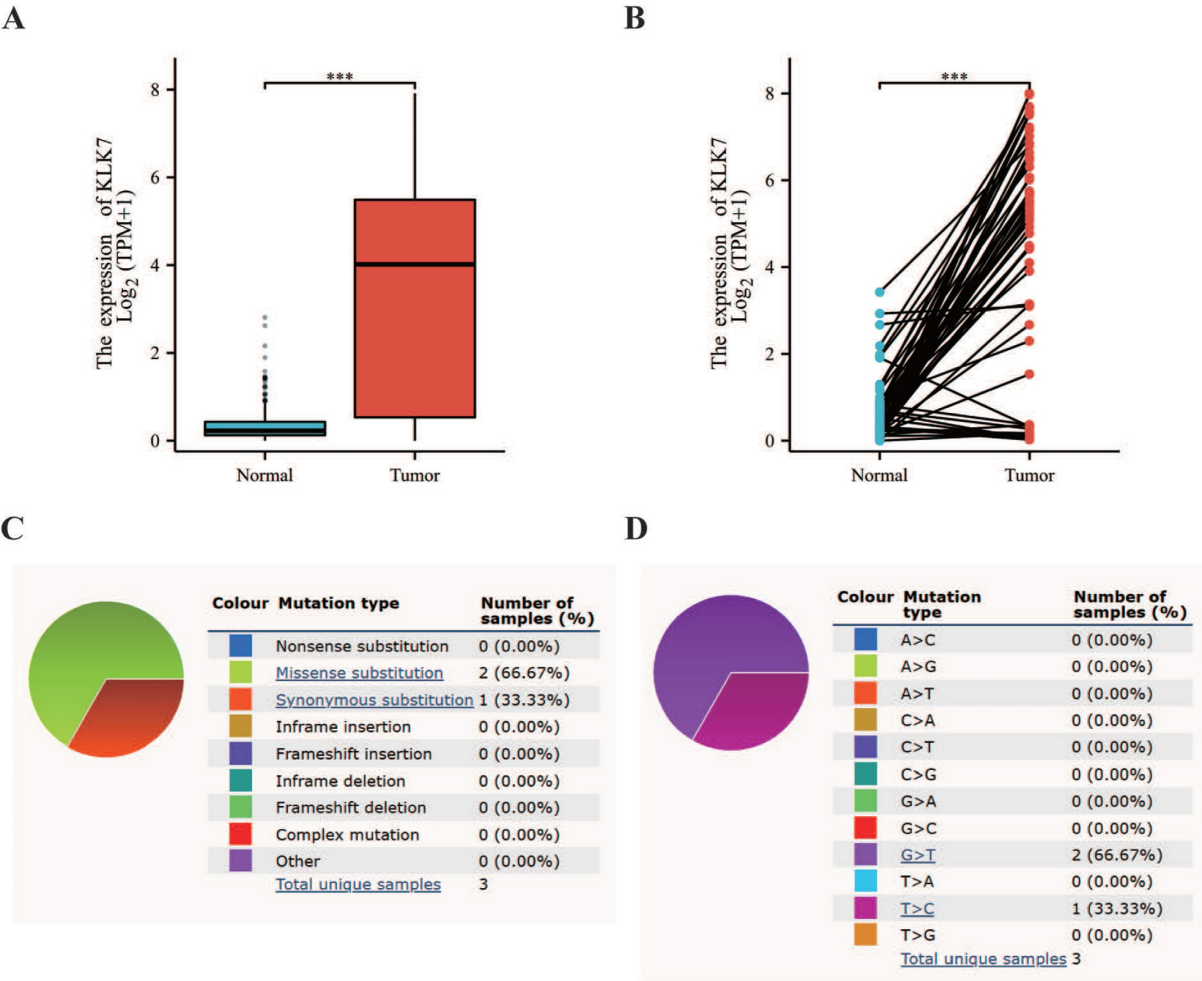
** Differential expression and mutation analysis of KLK7.** (A) Box plot of KLK7 expression differences in normal and tumor tissue in THCA. (B) Paired sample plot of KLK7 expression in THCA. (C-D) Pie charts of KLK7 mutation types. ***P < 0.001.

Utilizing the TCGA database, we discovered that KLK7 mRNA levels were elevated in THCA tissues when compared to paraneoplastic tissue (Figure 1A, Figure 1B). Additionally, mutation status was analyzed using data from the COSMIC database. The mutation status of KLK7 in thyroid cancer was shown in Figure 1C. Additionally, the base substitutions were primarily G > T (66.67%) and T > C (33.33%) (Figure 1D).

### 3.2. Enrichment analysis of KLK7 expression phenotypes

**Figure 2:**
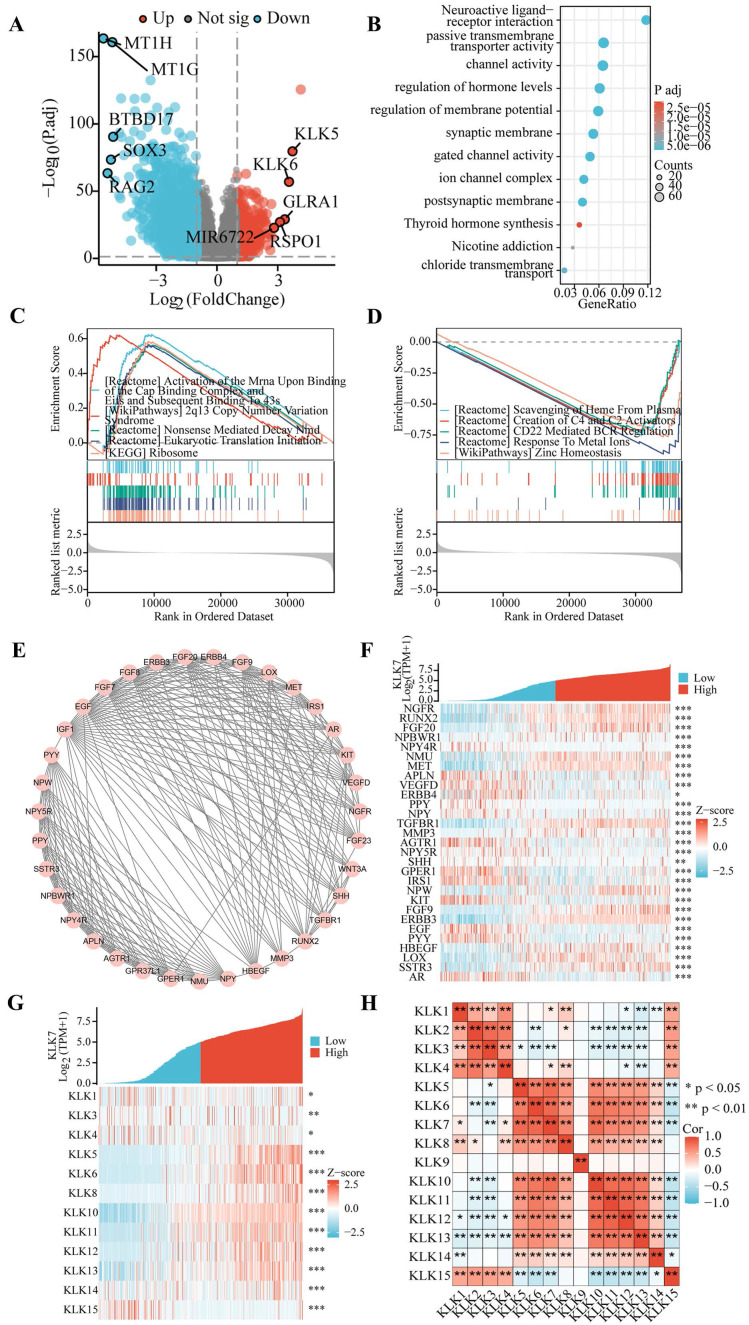
** Correlation and enrichment analysis of KLK7.** (A) Volcano plot of single-gene differential screening of KLK7, and the top ten genes exhibiting the most significant differences were marked. (B) Bubble chart of GO and KEGG analysis of related genes. (C-D) Classic visualization of GSEA analysis of related genes. (E) PPI network diagram of hub genes. (F) Co-expression heatmap of the top 29 hub genes. (G) Co-expression heatmap of the KLKs. (H) Correlation heatmap of the KLKs. *P < 0.05; **P < 0.01; ***P < 0.001.

Utilizing the TCGA database, we identified 2,934 genes with significant differential expression, highlighting notable differences between THCA samples with elevated and reduced KLK7 levels. The volcano plot (Figure 2A) demonstrates that 616 genes exhibit high expression levels in THCA samples with elevated KLK7, while 2,318 genes show low expression. Enrichment analysis of the 2,934 differentially expressed genes was conducted using GO and KEGG, identifying 12 relevant signaling pathways. Notably, these enriched pathways were primarily linked to passive transmembrane transporter activity, channel function, hormone regulation, membrane potential control, synaptic and postsynaptic membranes, gated channel activity, ion channel complexes, thyroid hormone synthesis, nicotine addiction, and chloride ion transmembrane transport (Figure 2B). Furthermore, to comprehensively explore KLK7-related pathways in THCA, GSEA analysis was also performed. The GSEA results indicated that in samples with high KLK7 mRNA expression, there was an enhancement in the regulation of protein synthesis, genetic variation, and mRNA degradation (Figure 2C), while certain pathways were inhibited, including heme clearance in plasma, generation of C4 and C2 activators, CD22-mediated B cell receptor regulation, response to metal ions, and zinc homeostasis (Figure 2D).

### 3.3. Examination of genes linked to KLK7 in thyroid cancer

Figure 2E illustrates that the MCODE plugin detected 35 hub genes associated with KLK7 within the PPI network. The top 29 genes correlated with KLK7 mRNA expression were illustrated in Figure 2F. Furthermore, the relationship between KLK7 mRNA expression and other KLK genes is depicted in Figure 2G. Ultimately, we discovered that the mRNA expression of most KLK genes was strongly associated with KLK7 mRNA expression (Figure 2H).

### 3.4. Analysis of the affiliation among KLK7 expression and clinical features in THCA patients

**Figure 3:**
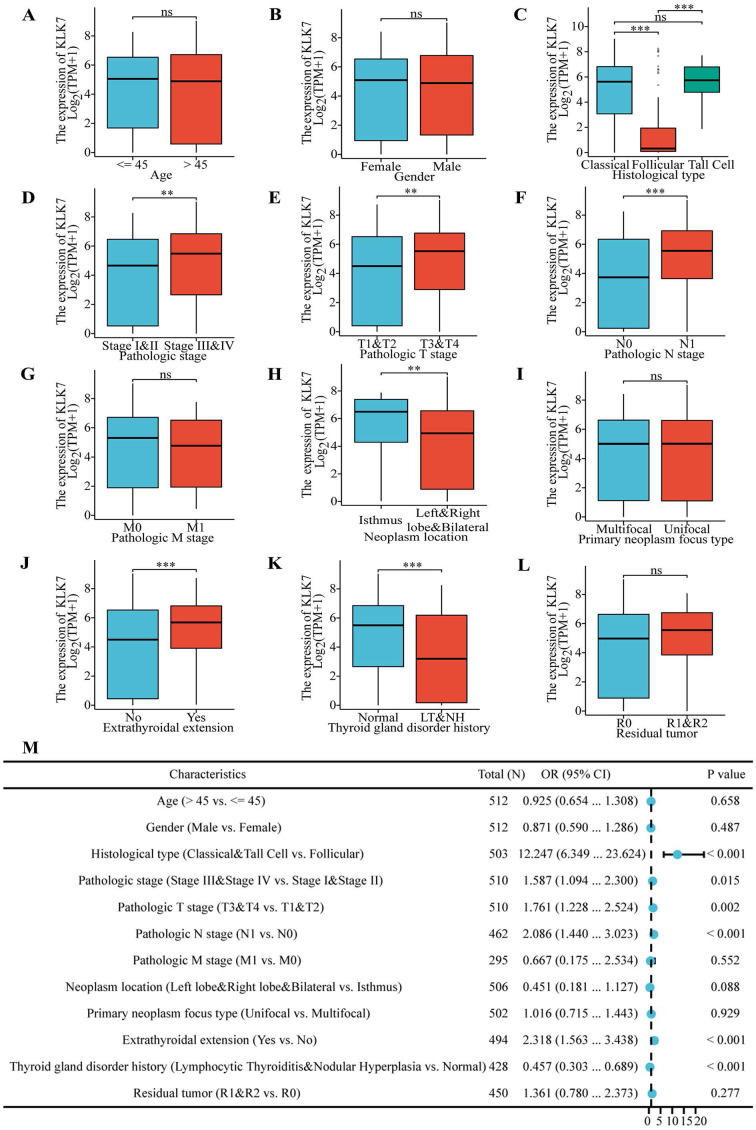
** Association of KLK7 expression with clinicopathologic factors.** (A-L) Differences in KLK7 gene expression in response to different clinical factors. (M) The risk factors for KLK7 mRNA high expression were shown. **P < 0.01; ***P < 0.001.

To elucidate the impact of KLK7 expression on the progression of THCA, clinical data from the TCGA database were analyzed to assess the association between KLK7 expression and the clinical characteristics. We discovered that KLK7 mRNA expression is linked to histological type (Figure 3C), pathological stage (Figure 3D), pathological T stage (Figure 3E), pathological N stage (Figure 3F), tumor location (Figure 3H), extrathyroidal extension (Figure 3J), and a history of thyroid gland disorders (Figure 3K). No notable correlation was observed among KLK7 mRNA expression and age (Figure 3A), gender (Figure 3B), pathological M stage (Figure 3G), primary tumor type (Figure 3I), or residual tumor (Figure 3L). In particular, KLK7 mRNA levels were significantly elevated in classical and tall cell variants, stages III and IV, T3 and T4, N1, isthmus tumors, extrathyroidal extension, and among patients without prior thyroid gland conditions, when compared to the control group. Additionally, logistic regression analysis indicated that classical & tall cell types, stages III & IV, T3 & T4, N1, extrathyroidal extension, and no history of thyroid gland disorders are risk factors for high KLK7 mRNA expression in THCA (Figure 3M).

### 3.5. Analysis of the correlation among KLK7 and diverse immune cells and immune checkpoints

**Figure 4:**
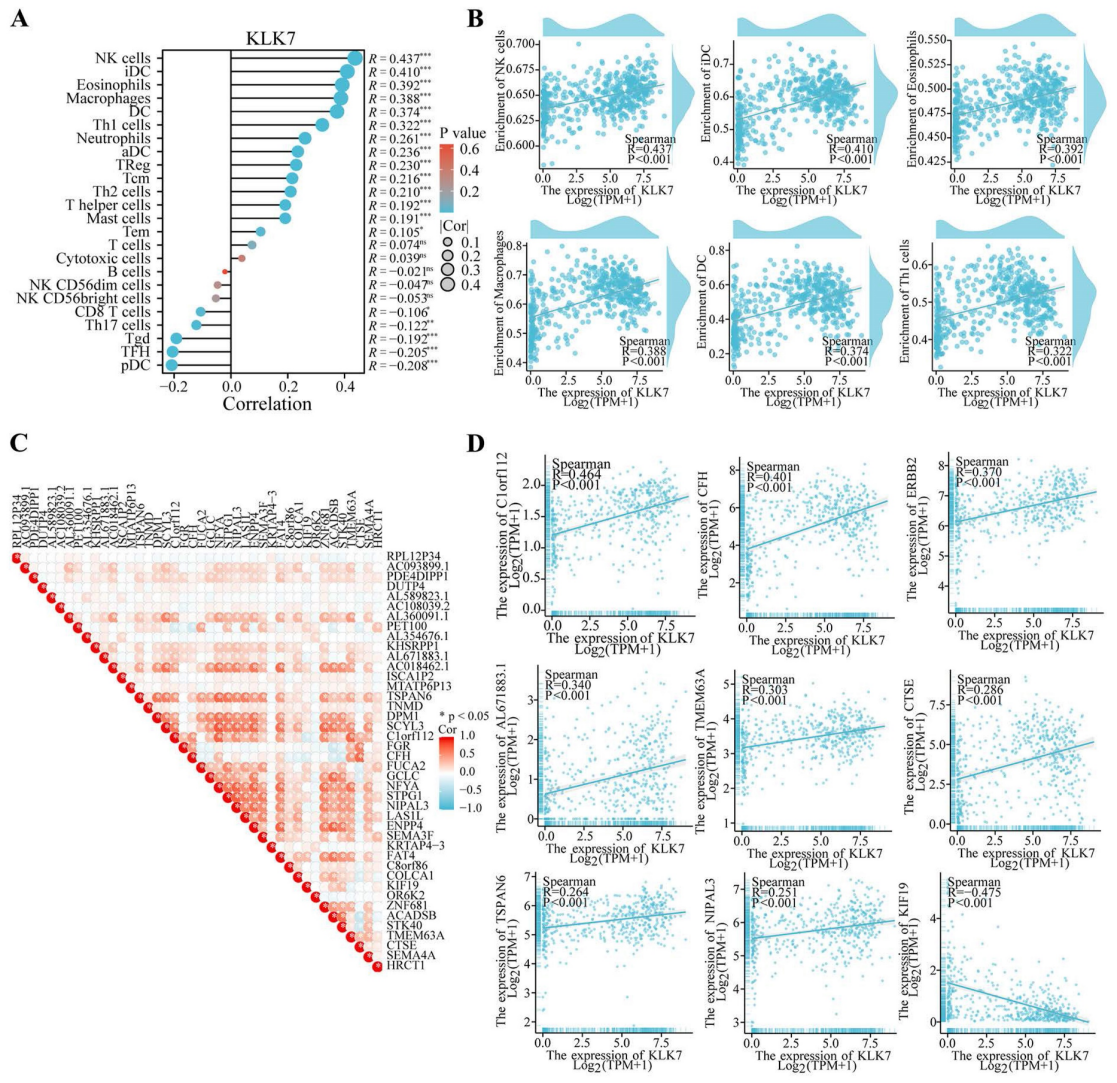
** Association of KLK7 with various immune cells and immune checkpoints.** (A-B) Correlation analysis of KLK7 expression with immune cells. (C-D) Correlation analysis of KLK7 expression with immune checkpoints. *P < 0.05.

To investigate the effects of KLK7 alterations on the tumor microenvironment, immune infiltration was analyzed using the ssGSEA technique. As shown in Figure 4, KLK7 was positively correlated with NK cell (R = 0.437, P < 0.001), iDC (R = 0.410, P < 0.001), eosinophils (R = 0.392, P < 0.001), macrophages (R = 0.388, P < 0.001), DC (R = 0.374, P < 0.001), and Th1 cells (R = 0.322, P < 0.001) (Figure 4A-B). And immune checkpoint analysis showed that KLK7 was positively correlated with C1orf112, CFH, ERBB2, AL671883.1, TMEM63A, CTSE, TSPAN6, and NIPAL3, and negatively associated with KIF19 (R = -0.475, P < 0.001) (Figure 4C-D).

### 3.6. KLK7 expression is elevated in PTC tissues and cells

**Figure 5:**
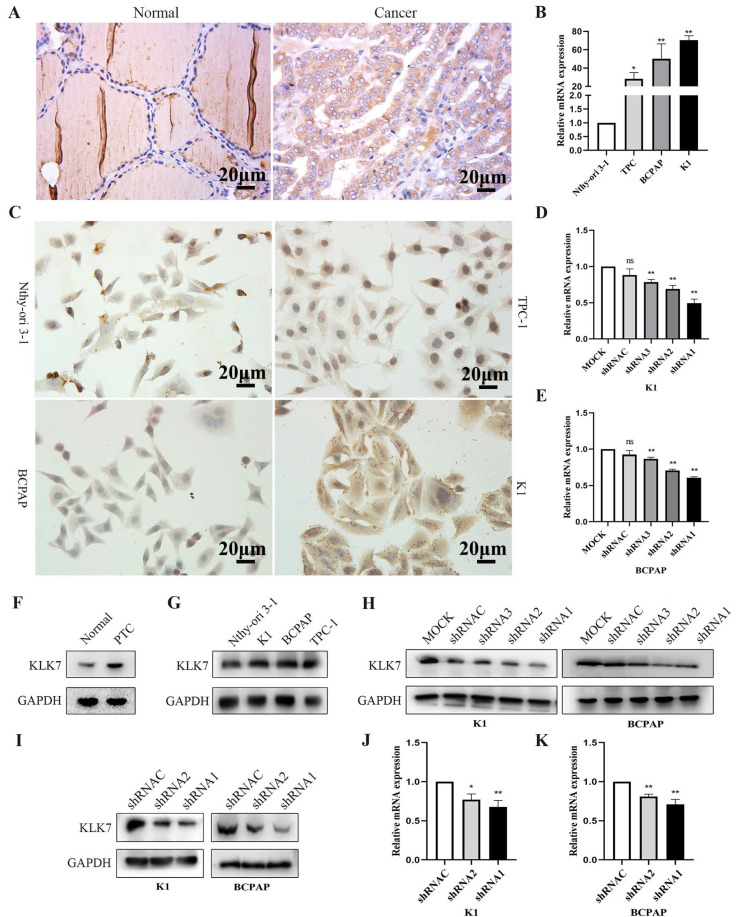
** KLK7 expression is increased in PTC.** (A) Immunohistochemical staining of KLK7 in PTC and normal tissues (Magnification: 400 ×; Scale bar: 20 μm). (B) RT-qPCR was used to analyze the expression of KLK7 mRNA in PTC cell lines. (C) The expression of KLK7 in PTC cell lines. (D-E) RT-qPCR was used to analyze the expression of KLK7 mRNA in K1 and BCPAP cells. (F) The protein expression of KLK7 in PTC and normal tissues. (G) Western blot was used to analyze the expression of KLK7 protein in PTC cell lines. (H) Western blot was used to analyze the expression of KLK7 protein in K1 and BCPAP cells. (I) Western blot analysis of KLK7 protein level in K1 and BCPAP cells transfected with KLK7-shRNAC, KLK7-shRNA2, and KLK7-shRNA1. (J-K) RT-qPCR to detect KLK7 mRNA level in K1 and BCPAP cells transfected with KLK7-shRNAC, KLK7-shRNA2, and KLK7-shRNA1. *P < 0.05; **P < 0.01.

The findings from immunohistochemistry indicated that KLK7 protein was predominantly localized in the cytoplasm, with positive signals appearing as brownish-yellow. KLK7 was overexpressed in PTC (Figure 5). And KLK7 expression levels were positively correlated with clinical stage (R = 0.65, P < 0.05) and lymphatic node metastasis (R = 0.64, P < 0.05) (Table 1). Western blot analysis also revealed that KLK7 expression was significantly higher in PTC tissues than in normal thyroid tissues (P < 0.05, Figure 5F).

As shown in Figure 5B-C and 5G, KLK7 was overexpressed in K1 and BCPAP cell lines compared with normal thyroid cells (P < 0.01). To study the biological functions of KLKs in PTC, we silenced KLK7 expression in PTC cell lines K1 and BCPAP, aiming to determine its potential impact. To observe the silencing efficiency of KLK7-shRNA1, KLK7-shRNA2, KLK7-shRNA3, and KLK7-shRNAC on K1 cells, RT-qPCR and WB analyses were executed. In comparison to the control group, KLK7 expression was markedly reduced in the KLK7-shRNA1 and KLK7-shRNA2 groups within K1, with similar findings observed in BCPAP (P < 0.01, Figure 5D-E, Figure 5H-K).

### 3.7. Functional verification of KLK7 in PTC

**Figure 6:**
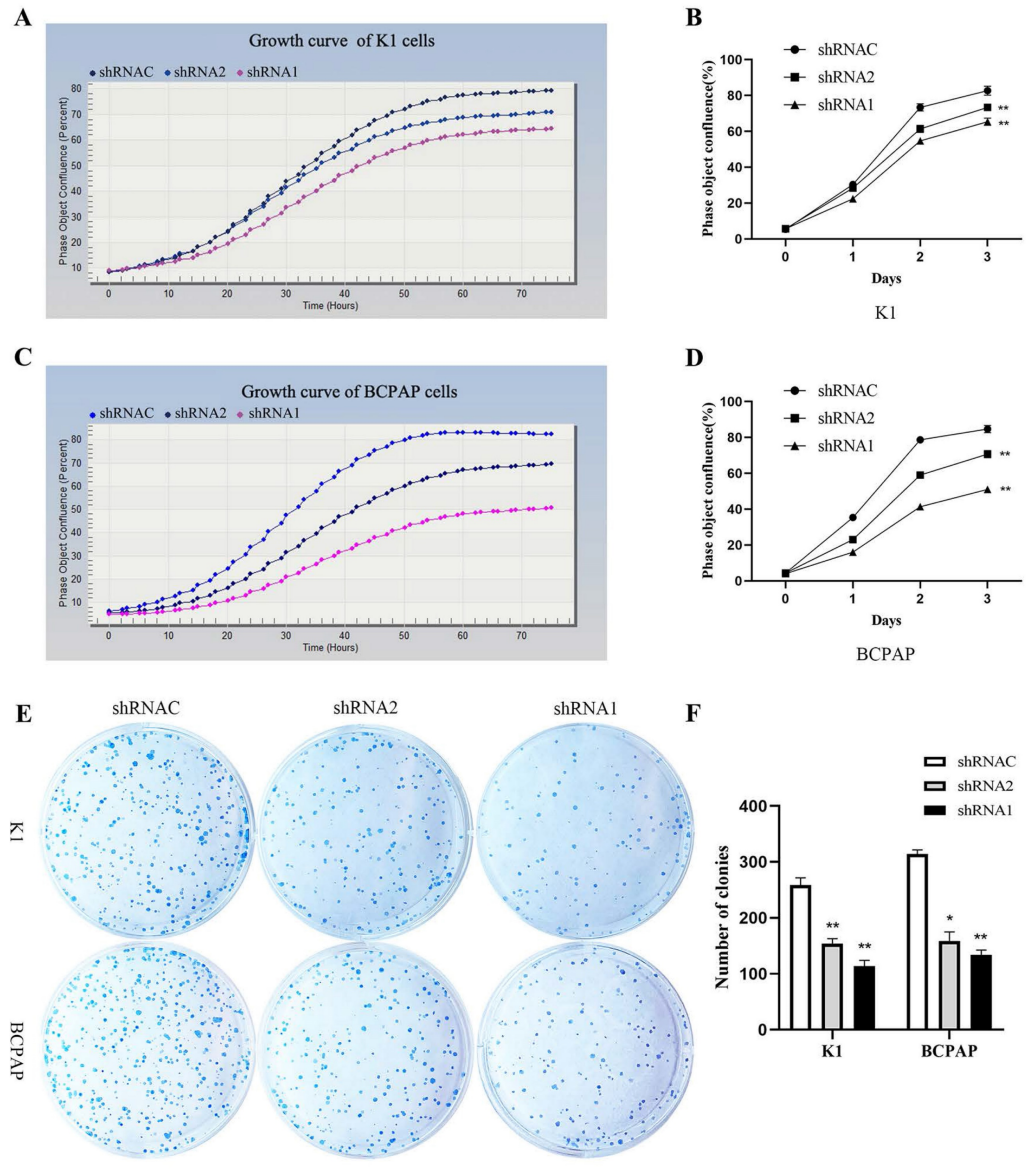
** Proliferation functional verification of KLK7 in PTC.** (A-B) Growth curve was used to detect the proliferation of K1 cells after KLK7 knockdown. (C-D) Growth curve was used to detect the proliferation of BCPAP cells after KLK7 knockdown. (E-F) Silencing of KLK7 decreased the clone formation ability of K1 and BCPAPA cells. *P < 0.05; **P < 0.01.

**Figure 7:**
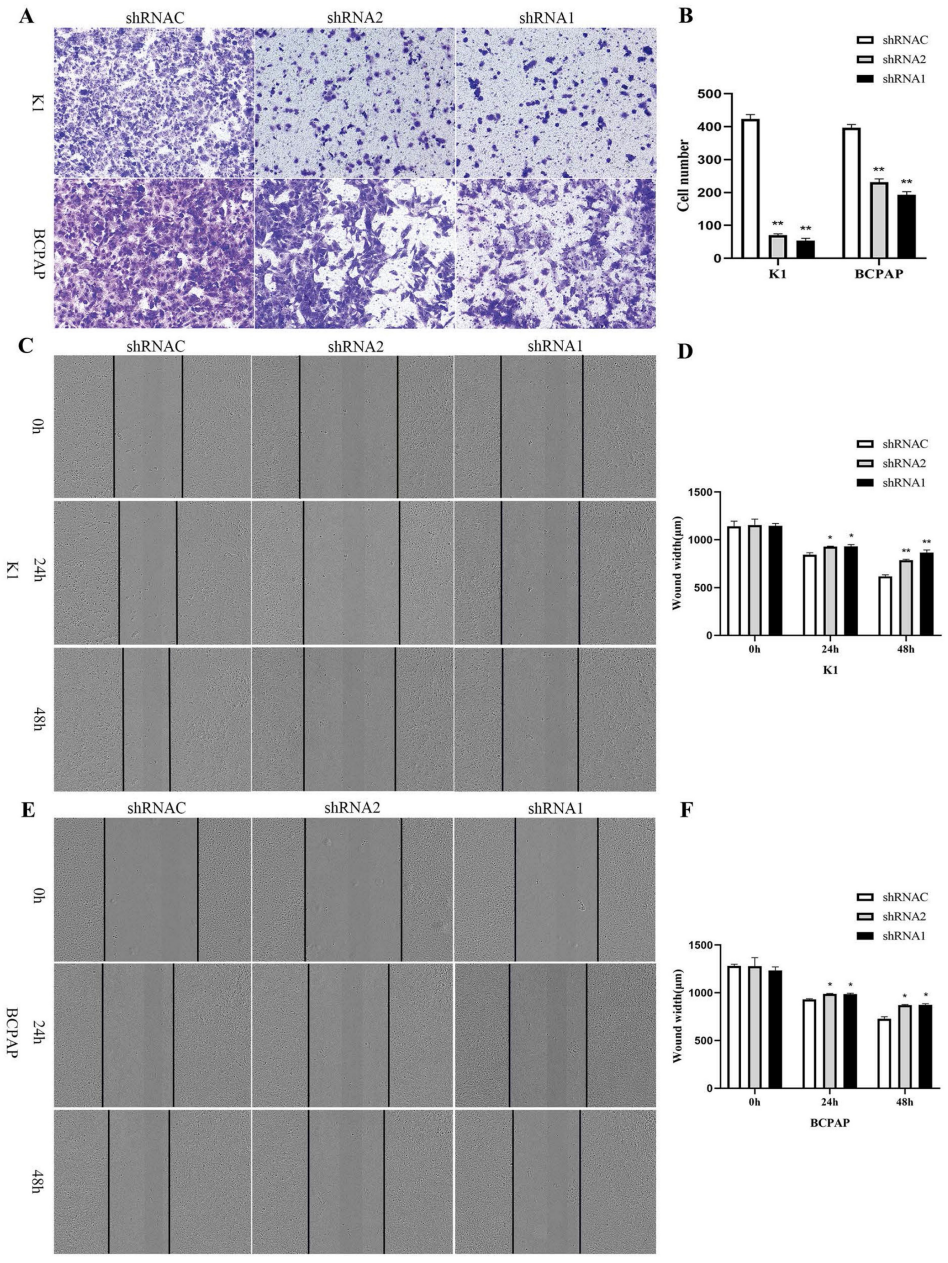
** Migration and invasion functional verification of KLK7 in PTC.** (A-B) Transwell assay was used to assess cell invasion capability. (C-F) Wound healing assay showed that KLK7 knockdown led to impairment in cell migration ability of K1 and BCPAP. *P < 0.05; **P < 0.01.

Growth curves showed that after silencing KLK7 expression, the KLK7-shRNA1/2 group had a significantly lower growth rate in comparison to the shRNA control group in K1 and BCPAP cells (P < 0.05, Figure 6A-D). Cell cloning experiments disclosed that the quantity of cell colonies significantly diminished within the KLK7-shRNA1/2 groups after silencing KLK7 expression. The decrease in colony count was notably significant when in comparison to the shRNA control group (P < 0.05, Figure 6E-F). The transwell invasion assay indicated a notable diminish within the quantity of cells traversing the membrane in the KLK7-shRNA group relative to the KLK7-shRNAC group, with the reduction being statistically significant (P < 0.05, Figure 7A-B). The findings from the wound healing assay indicated that silencing KLK7 resulted in a significant decrease in the migration rate of shRNA-KLK7 cells when compared to the KLK7-shRNAC group (P < 0.05, Figure 7C-F).

### 3.8. Effect of KLK7 silencing on EMT and MAPK pathway in PTC cells

**Figure 8:**
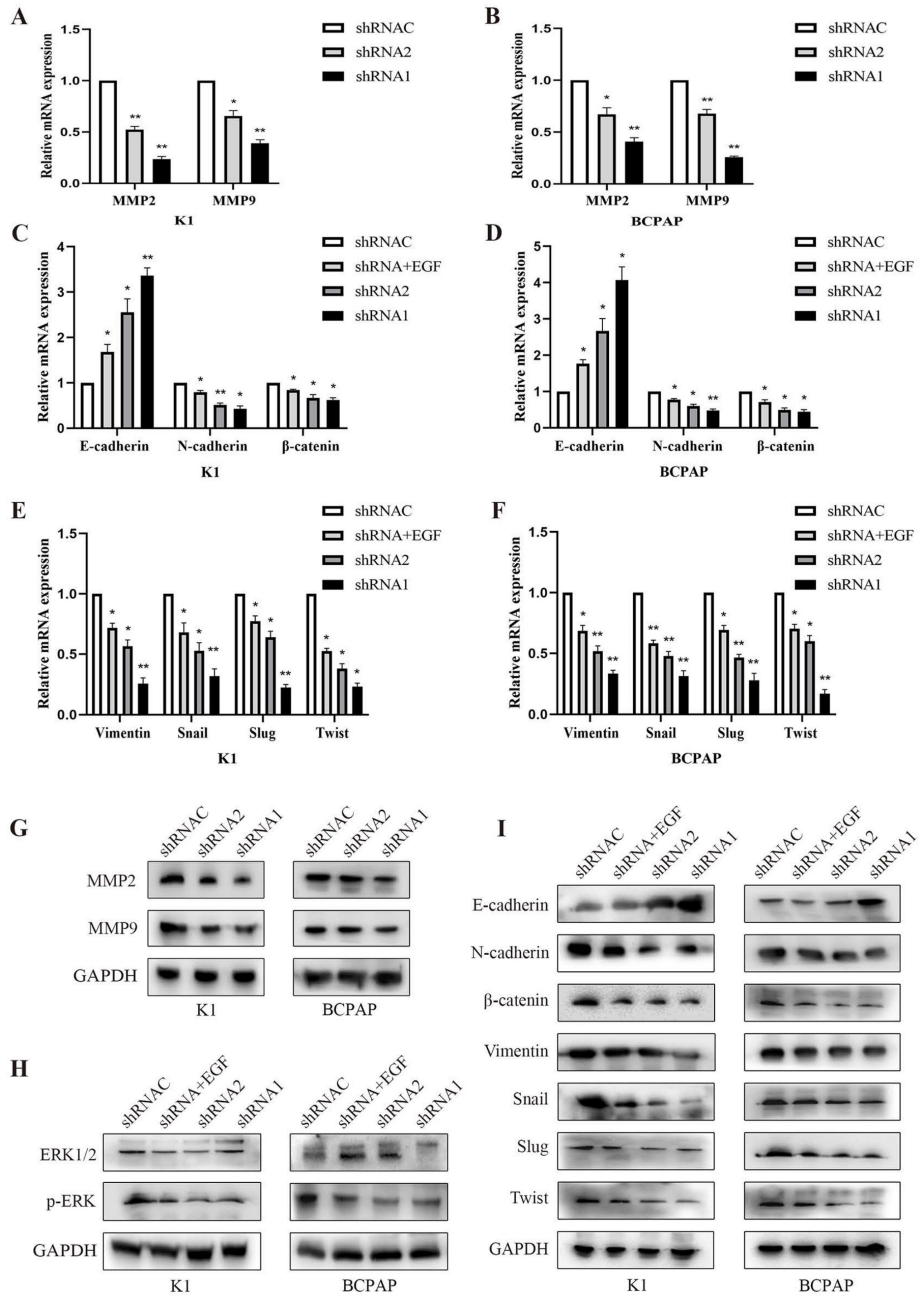
** Effect of KLK7 silencing on EMT and MAPK pathway in PTC cells.** (A-B) The MMP2 and MMP9 levels were measured by RT-qPCR. (C-F) RT-qPCR of differentially expressing EMT-related genes in K1 and BCPAP cells. (G) The MMP2 and MMP9 levels were measured by western blot. (H) The levels of ERK1/2 and p-ERK were measured by western blot. (I) Western blot analysis of EMT protein levels in K1 and BCPAP cells. *P < 0.05; **P < 0.01.

Following the suppression of KLK7 expression, both MMP2 and MMP9 mRNA and protein levels diminished, resulting in a lowered invasive capacity (P < 0.05, Figure 8A-B, Figure 8G). The WB analysis indicated that suppressing KLK7 expression resulted in reduced levels of the MAPK pathway protein p-ERK (P < 0.05, Figure 8H) and a reduction in EMT processes. Specifically, the epithelial marker E-cadherin was upregulated, while mesenchymal markers N-cadherin, Vimentin, and the transcription factors Snail, Slug, and Twist were significantly downregulated, along with β-catenin. Compared to the silencing group, the KLK7-shRNA + EGF group (50ng/mL) exhibited increased p-ERK expression, leading to an enhanced EMT process, characterized by a reduction in the epithelial marker E-cadherin and an increase in N-cadherin, β-catenin, Vimentin, Snail, Slug, and Twist levels (P < 0.05, Figure 8I). RT-qPCR results showed that inhibition of KLK7 caused upregulation of the epithelial marker E-cadherin and downregulation of N-cadherin, β-catenin, Vimentin, Snail, Slug, and Twist. Similarly, in the KLK7-shRNA + EGF group, p-ERK expression was elevated compared to the silencing group, promoting the EMT process, as indicated by downregulation of E-cadherin and upregulation of N-cadherin, β-catenin, Vimentin, Snail, Slug, and Twist (P < 0.05, Figure 8C-F).

### 3.9. Study on tumor development in immunodeficient mice

**Figure 9:**
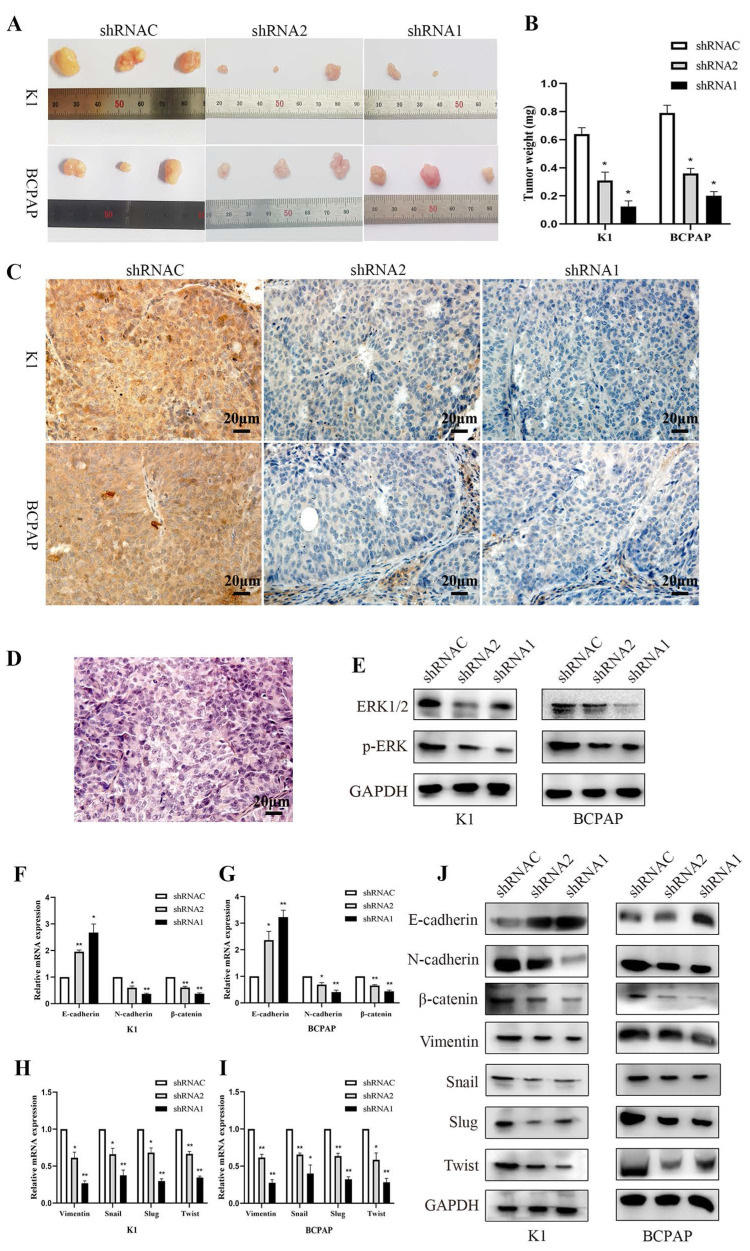
** Suppression of tumor growth by silencing the KLK7 gene in the K1 and BCPAP cells subcutaneous xenografts.** Three groups (KLK7-shRNAC, KLK7-shRNA2, and KLK7-shRNA1 groups) were subcutaneous xenografted onto nude mice. (A) The tumor growth of sh1-KLK7 and sh2-KLK7 groups were significantly inhibited compared with shNC-KLK7 group. (B) Mice were sacrificed after 28 days. The weights of tumors were weighed. (C) Immunohistochemical analysis of the expression of KLK7 in K1 and BCPAP cell subcutaneous xenograft tumors (400 ×). (D) Hematoxylin-eosin (H&E) staining of the subcutaneous xenograft tumors (400 ×). (E) WB analysis of ERK1/2 and p-ERK compared to GAPDH from tissue extracts. (F-I) RT-qPCR analysis of E-cadherin, N-cadherin, β-catenin, Vimentin, Snail, Slug, and Twist compared to β-actin from tissue extracts. (J) WB analysis of E-cadherin, N-cadherin, β-catenin, Vimentin, Snail, Slug, and Twist compared to GAPDH from tissue extracts. *P < 0.05; **P < 0.01.

In the tumor formation experiment with nude mice, we recorded the mice's weight gain and tumor development. The results demonstrated that silencing KLK7 inhibited the proliferation of BCPAP and K1 cells (P < 0.05, Figure 9A-B). The results showed a significant reduction in KLK7 expression levels in tumor samples from the KLK7 knockdown group (P < 0.05, Figure 9C-D). Western blot analysis revealed no significant difference in ERK1/2 levels in the KLK7-silenced group, but a reduction in p-ERK levels (P < 0.05, Figure 9E). Additionally, both RT-qPCR and WB results demonstrated increased protein expression of the epithelial marker E-cadherin, with decreased expression of the mesenchymal markers N-cadherin and Vimentin, as well as reduced expression of the transcription factors Snail, Slug, and Twist, along with a decrease in β-catenin levels. These findings are consistent with the results of *in vitro* experiments (P < 0.05, Figure 9F- J).

## 4. Discussion

KLKs have been discovered to enhance the growth and spread of cancer cells in multiple types of cancer 17. KLK7, a member of the KLK family, has attracted increasing research attention in recent years. Similar to other KLKs, KLK7 is significantly overexpressed in various malignancies, including ovarian cancer, breast cancer, and colorectal cancer 10, 11, 18. In such cancers, KLK7 facilitates tumor spread and metastasis by breaking down components of the extracellular matrix 19-22. We hypothesize that the overexpression of KLK7 mRNA and protein in PTC tissues may be driven by mechanisms associated with invasion and metastasis. Furthermore, to the best of our knowledge, no studies have yet investigated the role of KLK7 in the progression of PTC. Previous research from our group has suggested that the level of expression of KLK7 could be linked to clinical staging and poor prognosis. Although alterations in the mRNA, and protein levels of KLK7 have been observed in thyroid cancer, the specific mechanisms by which these changes influence thyroid cancer development, particularly in PTC, remain poorly understood.

To further investigate the impact of KLK7 alterations in THCA, we analyzed expression differences and mutations of KLK7 using data from the TCGA and COSMIC databases. Differential gene expression analysis based on TCGA data identified potential enriched pathways. Functional enrichment analysis suggested that KLK7 may play a role in positively regulating replication initiation and protein synthesis. Uncontrolled DNA duplication is a key feature of cancer cell growth, which is intimately linked to the process of protein production 23. Therefore, high expression of KLK7 may promote cell proliferation in THCA. To further validate this hypothesis, we performed a series of laboratory-based experiments. Cell experiments showed that silencing KLK7 effectively inhibited the proliferation of PTC cells, as indicated by growth curves and plate clone assays. This suggests that targeted inhibition of KLK7 could be beneficial for PTC patients. However, further experiments are needed to determine whether these effects are mediated through cell cycle inhibition or interference with DNA replication.

Bioinformatics analysis revealed that KLK7 mRNA levels are significantly associated with histological classification, clinical stage, tumor location, extrathyroidal extension, and a history of thyroid conditions in THCA patients, while showing no significant correlation with age or sex. Immunohistochemistry findings also indicated that KLK7 protein levels are strongly correlated with the clinical stage and lymph node metastasis in thyroid carcinoma patients. The results of these experiments indicate that KLK7 protein and mRNA levels are elevated in advanced thyroid cancer, suggesting that KLK7 could be a potential therapeutic target for this stage of the disease. In recent years, efforts have been made to develop KLK inhibitors as therapeutic agents. These inhibitors aim to block the proteolytic activity of KLKs, thereby impeding cancer progression and improving patient prognosis 24. Given KLK7's strong association with protein synthesis, we hypothesize that KLK7 inhibitors may offer significant potential in expanding the therapeutic options for THCA patients with poor prognosis.

Growing data indicates that specific low-molecular-weight inhibitors can alter the immunological landscape of tumor tissues and enhance immune-driven tumor cell destruction 25. Our gene function enrichment results suggest that KLK7 alterations may affect the immune microenvironment of THCA, including cellular immune response, inflammation and hypersensitivity reactions, immune surveillance, antigen processing, and immune initiation and regulation. Additionally, our findings suggest that KLK7 mRNA expression is either positively correlated with the proportions of specific immune cell populations. Immunological cells are a critical component of the tumor immunological microscopic environment, which may contribute to or hinder tumor development 26. Findings on immune infiltration imply that KLK7 could lower the levels of CD56 bright NK cells and plasmacytoid dendritic cells. CD56 bright NK cells represent a less mature subset of NK cells that possess antitumor properties 27. Concurrently, cancer patients exhibit a dysfunctional state of tumor-infiltrating plasmacytoid dendritic cells 28. These findings collectively suggest that combining KLK7 inhibitors with immune checkpoint inhibitors could be explored as a strategy to modulate the tumor immune microenvironment and enhance the efficacy of immunotherapy in PTC patients.

To delve deeper into how KLK7 influences PTC development, we chose two PTC cell lines, BCPAP and K1, and performed experiments including RT-qPCR and WB analysis. The experimental results demonstrated that reducing KLK7 expression significantly impaired migration and invasion capabilities, confirming its role as a crucial mediator of tumor progression. MMP2 and MMP9 are pivotal in the breakdown of the extracellular matrix and the spread of cancer, suggesting that KLK7 might control these enzymes to enhance the invasiveness of cancer cells 29, 30. Furthermore, the reduction in ERK1/2 phosphorylation levels following KLK7 silencing highlighted the involvement of the MAPK/ERK pathway, a pivotal signaling pathway in oncogenesis and advancement 31. Concurrently, changes in EMT markers further confirmed the role of KLK7 of PTC. Knockdown of KLK7 resulted in an increase in the epithelial marker E-cadherin, while levels of mesenchymal markers (N-cadherin, Vimentin), transcription factors (Snail, Slug, Twist), and β-catenin were reduced, indicating a reversal of EMT. Epidermal growth factor (EGF), one of the first identified growth factors, can activate the Ras-Raf-MEK-ERK pathway through epidermal growth factor receptor (EGFR), thereby promoting EMT 32. EGF is a classic activator of the ERK pathway and has been shown to induce EMT in various tumors, enhancing cell migration and invasion 33. The addition of the shKLK7+EGF group further validated that KLK7 may function as an upstream regulator of EMT, facilitating thyroid cancer cell metastasis by promoting the MAPK/ERK signaling pathway and coordinating the EMT process.

However, it is important to note that, although the expression of KLK7 in PTC was confirmed through bioinformatics analysis and related experiments, certain limitations still remain. On one hand, the amount of tissue samples used in this experiment was relatively limited. On the other hand, this study focused on only three PTC cell lines; subsequent studies should include a broader range of thyroid cancer cell lines.

## 5. Conclusion

In conclusion, KLK7 might mainly contribute to the growth, movement, and invasiveness of PTC, potentially influencing the EMT process via the MAPK/ERK pathway, thus impacting PTC development.

## Supplementary Material

Supplementary table.

## Figures and Tables

**Table 1:** Relationship between KLK7 and the clinical pathological parameters of PTC.

Characteristics	Total	KLK7 expression		*P*	r
		Low	High		
Gender					
male	11	8	3	0.488	
female	27	16	11		
Age(years)					
<55	10	8	2	0.268	
≥55	28	16	12		
Clinical stage					
Ⅰ+Ⅱ	30	22	8	0.034	0.65
Ⅲ+Ⅳ	8	2	6		
Tumor size(cm)					
<2.0	17	13	4	0.181	
≥2.0	21	11	10		
Lymph node metastasis					
negative	32	23	9	0.018	0.64
positive	6	1	5		
