# Supplementary material for: KLK7 Involvement in Thyroid Papillary Carcinoma Cell Migration and Invasion by EMT via MAPK/ERK Pathways
Source: J Cancer. 2025 Feb 11;16(5):1709–25. doi: 10.7150/jca.101555 (PMC11843248; doi:10.7150/jca.101555)
Supplement: Supplementary file 1 — Supplementary table. [file jcav16p1709s1.pdf]

Gene	Primer sequence
β-actin	F: 5′-ACTGGAACGGTGAAGGTGAC-3′
	R: 5′-AGAGAAGTGGGGTGGCTTTT-3′
KLK7	F: 5′-TTAGCCTTGGAAACTGCAGGA-3′
	R: 5′-TGCACGGTGTACTCATTCATC-3′
MMP2	F: 5′-ATCCTGGCTTTCCCAAGCTC-3′
	R: 5′-CACCCTTGAAGAAGTAGCTGTG-3′
MMP9	F: 5′-GGGCTTAGATCATTCCTCAGTG-3′
	R: 5′-GCCATTCACGTCGTCCTTAT-3′
E-cadherin	F: 5′-GAATGACAACAAGCCCGAAT-3′
	R: 5′-GACCTCCATCACAGAGGTTCC-3′
N-cadherin	F: 5′-TGCGGTACAGTGTAACTGGG-3′
	R: 5′-GAAACCGGGCTATCTGCTCG-3′
β-catenin	F: 5′-GGCCTCTGATAAAGGCTACTGTTG-3′
	R: 5′-ACGCAAAGGTGCATGATTTG-3′
Vimentin	F: 5′-CAGGCAAAGCAGGAGTCCAC-3′
	R: 5′-GCAGCTTCAACGGCAAAGTTC-3′
Snail	F: 5′-GCTGCAGGACTCTAATCCAGA-3′
	R: 5′-ATCTCCGGAGGTGGGATG-3′
Slug	F: 5′-GCTACACAGCAGCCAGATTCC-3′
	R: 5′-AACAGAGCATTTGCAGACAGGTC-3′
Twist	F: 5′-CATGTCCGCGTCCCACTAG-3′
	R: 5′-TGTCCATTTTCTCCTTCTCTGG-3′

Table 1. The primer sequences used in this study.
